# Combining synchrotron radiation techniques for the analysis of gold coins from the Roman Empire

**DOI:** 10.1038/s41598-022-19682-8

**Published:** 2022-09-23

**Authors:** I. Carlomagno, P. Zeller, M. Amati, G. Aquilanti, E. Prenesti, G. Marussi, M. Crosera, G. Adami

**Affiliations:** 1 Elettra - Sincrotrone Trieste, Trieste, 34149 Italy; 2 Dipartimento di Chimica, Università di Torino, Torino, Italy; 3 Dipartimento di Scienze Chimiche e Farmaceutiche, Università di Trieste, Trieste, Italy; 4 Present Address: Helmholtz-Zentrum Berlin für Materialien and Energie GmbH, BESSY II, Berlin, Germany; 5 Present Address: Dept. Inorganic Chemistry, Fritz-Haber-Institut der Max-Planck-Gesellschaft, Berlin, Germany

**Keywords:** Imaging studies, Characterization and analytical techniques, Imaging techniques, Microscopy

## Abstract

Four gold coins minted in the V century have been studied with non-destructive synchrotron radiation techniques, namely X-Ray Fluorescence (XRF) and X-ray Absorption Near Edge Spectroscopy (XANES). XRF data analyzed coupling standard and statistical methods were used to distinguish the composition of the alloy constituting the coins from that of successive deposits processes. Our analysis presents a quantification of the trace elements present in the metallic alloy providing interesting details for historical insight. Furthermore, on the basis of the XRF maps, some regions of interest were selected for XANES at the K-edge of Fe. Our analysis of the Fe spectra points out two main phases which can be related to Fe oxides naturally present in soil. From the relative abundance of these oxides, information on the site where the coins were found can be obtained, providing additional information on their fate across the centuries.

## Introduction

X-rays, being a non-destructive probe, are an excellent tool for the investigation of delicate, fragile, and valuable samples. This is certainly the case of ancient gold coins which, besides the intrinsic value due to the precious materials they are made of, have also a high historical worth.

Among the X-ray based techniques, X-Ray Fluorescence (XRF) is widely known in the cultural heritage community as an efficient way to analyse elements distribution and quantification^1^. Synchrotron Radiation (SR) sources, offering high photon flux and energy tunability, are suitable for achieving a superior degree of accuracy in XRF trace elements analysis with respect to laboratory sources^2^. However, this hardly compensates for the relatively difficult access to the facilities (long proposal preparation process and low acceptance rates) and for the limited time available for measurements, which prevents from analysing a high number of pieces typically involved in numismatics studies. On the other hand, SR offers the possibility of carrying out XRF at different energies, which can be useful to enhance the sensitivity to certain elements and to shed light on identifying the presence of a given element in case of emission lines overlap. A far more interesting advantage of SR is that many beamlines are capable of applying different techniques at once permitting a multimodal and full characterization of the samples in the same conditions. This is the case of the XRF beamline at Elettra synchrotron in Trieste, Italy^3^, where we have performed not only XRF, but also X-ray Absorption Near Edge Spectroscopy (XANES) measurements. XANES, among other information, assesses the local chemical environment and oxidation states of the elements which has been proven relevant for a wide variety of applications in cultural heritage^4^ .

The aim of this work is to describe a methodological strategy based on advanced analytical synchrotron techniques coupled with a statistical data analysis approach to find minor differences and give useful information to the historians on several aspects of ancient civilizations. On the XRF maps, collected with a spatial resolution of 100 $\mu$m, we apply a statistical analysis to disentangle the contributions coming from the metallic alloy from those due to the overlayer. In that way, not only do we obtain information on the metallic alloy, but we also have an insight on the accumulated debris, yielding details ranging from the Au minting process, to the source of the gold, to the area of conservation of the artefacts. On the basis of the XRF maps, a few areas of the coins were selected for XANES measurements at the Fe K-edge. From the investigation of the dirt accumulated in the hollow regions, we could characterize the Fe compounds deposited during the burial period. Through the identification and quantification of Fe oxides present in the sample, the burial area could be hypothesized based on the environmental conditions corresponding to the oxides presence in soils^5^.

## Results

### XRF analysis

In this section, we present the results obtained on one sample, but the same procedure described here was applied to all of them.

**Figure 1:**
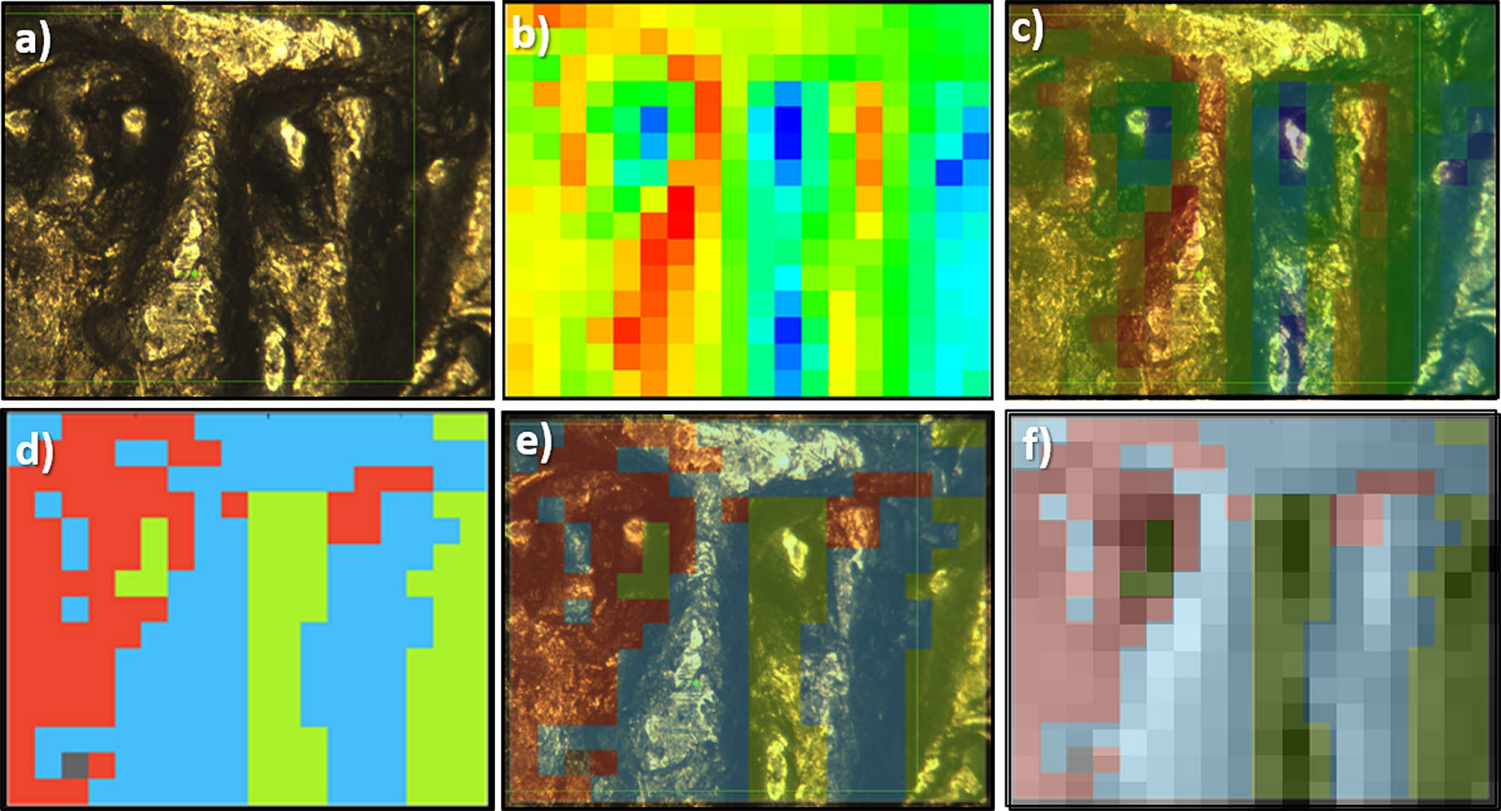
**(a)** Optical image of coin B, where the XRF map was collected, **(b)** fluorescence emission induced with an exciting beam of 10 keV in the area shown in panel *a*: the emission energy range corresponds to the Au M lines, the intensity follows a temperature scale, **(c)** superposition of panels (**a**) and (**b**), **(d)** the three groups found in the t-SNE analysis: blue for G1, red for G2, green for G3, **(e)** superposition of panel (**a**) and (**d**), and **(f)** superposition of panel *d* and a grey scale version of (**b**).

The XRF measurements were collected in a small region (about $2 \times 2 \mathrm{mm}^2$) of the obverse of the coins (for a detailed description of the samples, see “Samples” section, for the XRF maps of the coins, see the Supplementary materials, Fig. S1). For each coin, the area selected for the XRF map was raster scanned collecting the total fluorescence emission for each pixel (See Fig. 1a, b and c). The cumulative XRF spectrum is obtained by summing up the contribution of all the pixels. From the XRF cumulative spectrum of each coin, we could identify the emission peaks of several elements reported in Table 1.

The beam size with which the XRF maps were collected 100 x 100 $\mu m^2$ is a good compromise to achieve fast measurements with a spatial resolution being good enough to correlate the elements distribution with the topography of the coin. In addition, their spatial distribution contains the key information to distinguish the evenly-distributed components of the metal alloy from the materials deposited on the coin surface during the burial period, accumulated in indented areas. To classify each element in one of the two categories based on the topography, we used the t-distributed stochastic neighbour embedding (t-SNE) algorithm^6^ available in the Orange software^7^ (see “t-SNE” section).

**Table 1:** Elements identified by XRF analysis divided between metallic alloy and dirt.

Alloy	Dirt
Pd, Ag, Pt	Mg, Al, Si
Au, Hg, Pb	P, S, Cl, K
	Ca, Ti, V
	Cr, Mn, Fe
	Ni, Cu, Zn

**Figure 2:**
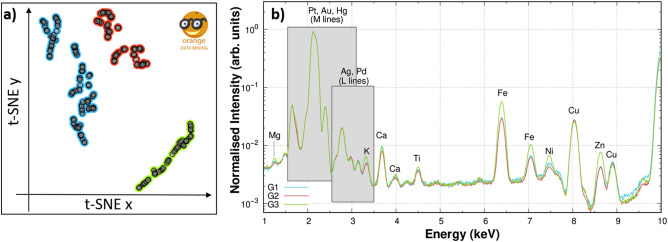
**(a)** t-SNE plot of the XRF map obtained with 10 keV on coin B. Three groups are identified: blue G1, red G2, and green G3. **(b)** The spectra of the three groups collected with an incident energy of 10 keV are shown upon normalisation to the Au emission peak at 2.1 keV. Each peak is identified with the element giving rise to that spectral feature.

The t-SNE analysis returned the plot shown in Fig. 2a in which three distinct groups could be identified: G1 (blue points), G2 (red points), and G3 (green points). G1 and G2 are both located in the top left part of the plot and are closer to each other than to G3, lying in the bottom right part of the plot instead. The fluorescence spectra corresponding to the pixels of the same group were summed up and are shown upon normalization to the Au emission peak at 2.1 keV in Fig. 2b. Noticeably, two of the three spectra (G1 and G2) are rather similar to each other, suggesting that they could be considered as a single group.

As a further element to validate this choice, their spatial distribution was considered. It is presented in Fig. 1d and for sake of comparison it is shown overlapped with the optical image (Fig. 1e) and with a grey scale version of the XRF map (Fig. 1f). The different morphology of the three groups is evident: while G1 and G2 are associated with mostly flat areas (red and blue pixels), the other covers mostly indented areas (green, G3).

This is consistent with the different spectral features in Fig. 2b: the G3 group (green) presents a particularly high intensity in the region of K, Ca, Ti, Fe, Ni, Zn peaks. These elements, compatible with soil composition, can be attributed to the debris accumulated in the hollows, therefore the spectra in this group were not considered for the evaluation of the alloy composition.

The advantage of using t-SNE analysis with respect to an arbitrary manual selection of the pixels evaluated as the best representation of the metallic alloy is that a higher number of pixels is selected on the basis of their spectral similarity. Conversely, one would have to rely only on position-based discrimination, assuming that the safest choice includes only the pixels in the flat, even regions. The t-SNE algorithm, based on statistic-evaluated similarity, groups together a higher amount of pixels, providing better resolved spectral features and, hence, higher accuracy results.

Once the pixels distinction was done, the same pixels were selected in the 11.6 keV map and a single spectrum was obtained summing up the contribution of the “gold alloy” groups, G1+G2. This spectrum was then analysed using PyMCA (see “PyMCA” section) obtaining the results reported in Table 2.

**Table 2:** Average date of coinage, and concentrations (%) obtained from the best fit parameters for XRF spectra at 11.6 keV relative to the G1+G2 regions of the four coins.

Sample	A	B	C	D
Period (AD)	409	409	417	440
Au	97.8	96.2	96.2	96.1
Ag	0.1	0.4	0.3	0.8
Pt	0.1	1.0	0.9	0.7
Hg	0.1	0.5	0.6	0.5
Pd	0.2	0.2	0.2	0.2
Pb	1.0	1.1	1.1	1.2

We have to stress here that due to the large uncertainty associated with the parameters of L and M emission lines^8,9^, our analysis is to be considered as a semi-quantitative one. This implies that, while our results do provide insightful details on the variations among the pieces, a direct comparison with data available in literature should be avoided. This approach, far from being unreliable, is already known in literature^10^ and represents a viable option to compare samples characterized in the same conditions. In particular, although the absolute values cannot be directly compared to those obtained in other experiments (i.e. under different conditions), the purity of the pieces can be reliably compared below 0.5%. The main limitation of our analysis is due to the fact that the the database available for L-shells calculations is affected by large uncertainties: this affects the absolute concentrations which might be off by 0.2-0.5%. However, even within this broader range, our results of Au concentrations are in agreement with the available literature^11^.

### Coins fineness

One of the most studied parameters of ancient coins is their fineness, as this can provide information on economic inflation or debasement dynamics in the ancient coinage^12^. However, preliminary XRF analysis carried out with laboratory sources showed no significant differences among the pieces. This agrees with the fair amount of literature already demonstrating how the Au fineness in Roman coins was around 98%^12,13^. Our results show that, except sample A, which is 97.8% pure, the others have a fineness around 96%, with slightly higher amounts of Hg, Ag, and Pt compensating for the missing Au. Considering the uncertainty of the absolute value around 2% (as discussed in “XRF analysis” section), we can say that the true value is in the range 94-98%. This range was already discussed in literature and is unlikely due to any intentional debasement but, most probably, is related to loose control on the gold sources. Such sources could be old coins or jewelry, resulting into similar fineness values (like the *aurei* issued by Cornuficius in I century BC^12^).

Once again, we stress that although we have analyzed a limited number of samples, our analysis is aimed at presenting the potentiality of a new approach based on the combination of different, complementary techniques. In particular, we combine XRF with a fine statistical analysis, trace elements evaluation, and XANES-based oxidation state study.

The quantification of trace elements can represent evidence for different supplies of gold^14^. Identifying sources of contaminants containing these fingerprints elements is crucial for an archaeometric analysis and cannot be overlooked, however, the analysis of elements like Pt, Hg, and Pb, requires some additional care. As the fluorescence lines of these elements overlap with Au M-lines, In the case of Pt, the L emission lines are found at 2050 eV, i.e. very close to the M emission lines of Au at 2122 eV. Alternatively, to address the issue regarding the other elements, a reference spectrum collected on a pure Au foil was used. This spectrum was subtracted from the spectra of each coin. As shown in Ref^15^ for the case of Pt traces in Au, upon the subtraction, the spectra were showing the “hidden” features, i.e. peaks which were previously unresolved due to the high intensity of the Au peaks, supporting the presence of these elements. The three spectra (coin, Au reference, subtraction) are shown in the Supplementary materials, in Fig.S2.

The presence of Pt can be interpreted as a fingerprint of the Au source^14,16^. In fact, Au can be extracted from the so-called primary deposits (i.e. naturally found within rock formations) or from secondary deposits, produced via weathering from the primary ones^17^. Pt is very rare in primary deposits and is commonly associated with secondary deposits as a result of fluvial transport^18^. Hence, the Pt/Au correlation can be used to identify different sources of gold in ancient artefacts^19^. Our results, in agreement with literature^14^, the amount of Pt was found to be $<0.5\%$ in coin A, and between 0.5% and 1.0% for all the others coins, suggesting a different origin of the gold used to struck coin A with respect to the others^19^. The fact that Au used for coin A might have a different origin is compatible with the higher purity of this sample: in fact, the small variation observed in the fineness ($\simeq 2\%$) is typically explained with better sources of Au^12^. Interestingly, coins A and B show differences on the basis of both the fineness and the Pt/Au correlation. These two coins were issued in the same period but in two different mints. Coin A presents an engraving “COM”, standing for *Comes Sacrarum Largitionum* which was the *comitatus* responsible for issuing *solidi* of fixed weight and fineness^13^. Coin B, on the other hand, presents another mint mark on its reverse, “CON”, which refers to the Constantinople mint.

### Debris analysis

**Figure 3:**
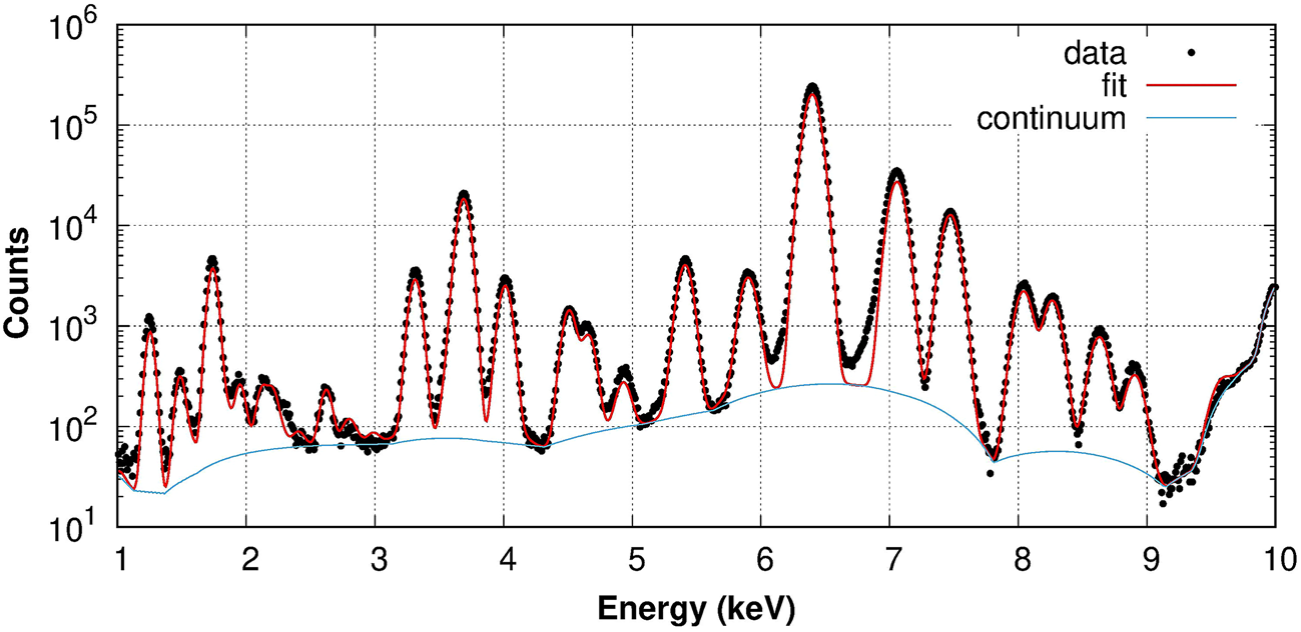
XRF spectrum collected using an incident beam of 10 keV (points) collected over 300 s in a hollow region of coin D, best-fit (thick red line) and continuum baseline used for the fit (thin blue line).

XRF analysis can also be employed to assess information about the debris composition. This can be useful to shed light on the nature of some encrustations found the artefacts^20^, or, like in our case, when dealing with dirt accumulated during burial periods. To understand the nature of such deposit, we collected a spectrum in a debris-rich area. The spectrum, shown in Fig. 3, was fitted using the multilayer algorithm implemented in PyMCA which allows to determine the thickness of the layers. Two layers were considered to represent the dirt and the coin. The coin composition was obtained from the above mentioned analysis and the same values of Table 2 were used. On the contrary, the debris composition was allowed to vary, as the deeper layer accumulated in the hollow area could be different from the shallower patina present on the even region. The initial guess, however, was considered to be equal to the contaminants found in the clean area. From this starting point, the thickness of the debris layer and its composition were varied until a good agreement with the experimental data was reached. The final result is shown in Fig. 3. The concentrations of the elements $>1\%$ are reported in Table 3; traces of Ni, Cr, Mn, Ti, Cl, Cu, P, Zn were also found. The debris layer is found to be approximately 4.3 $\mu$m thick. The light elements (like O and C) are not reported because the cross section of their X-ray absorption is fairly low at 10 keV, and their fluorescence is easily shielded by the other elements before reaching the detector.

**Table 3:** Concentrations (%) of the main elements making up the debris accumulated in the hollow region of coin D. The values were obtained from the best fit of a single XRF spectrum collected on the same spot for 300 s. Uncertainties are $\pm 0.5\%$.

Element	Concentration (%)
Si	31.0
Fe	26.6
Mg	23.0
Ca	9.0
Al	5.0
K	2.0

### XANES analysis

**Figure 4:**
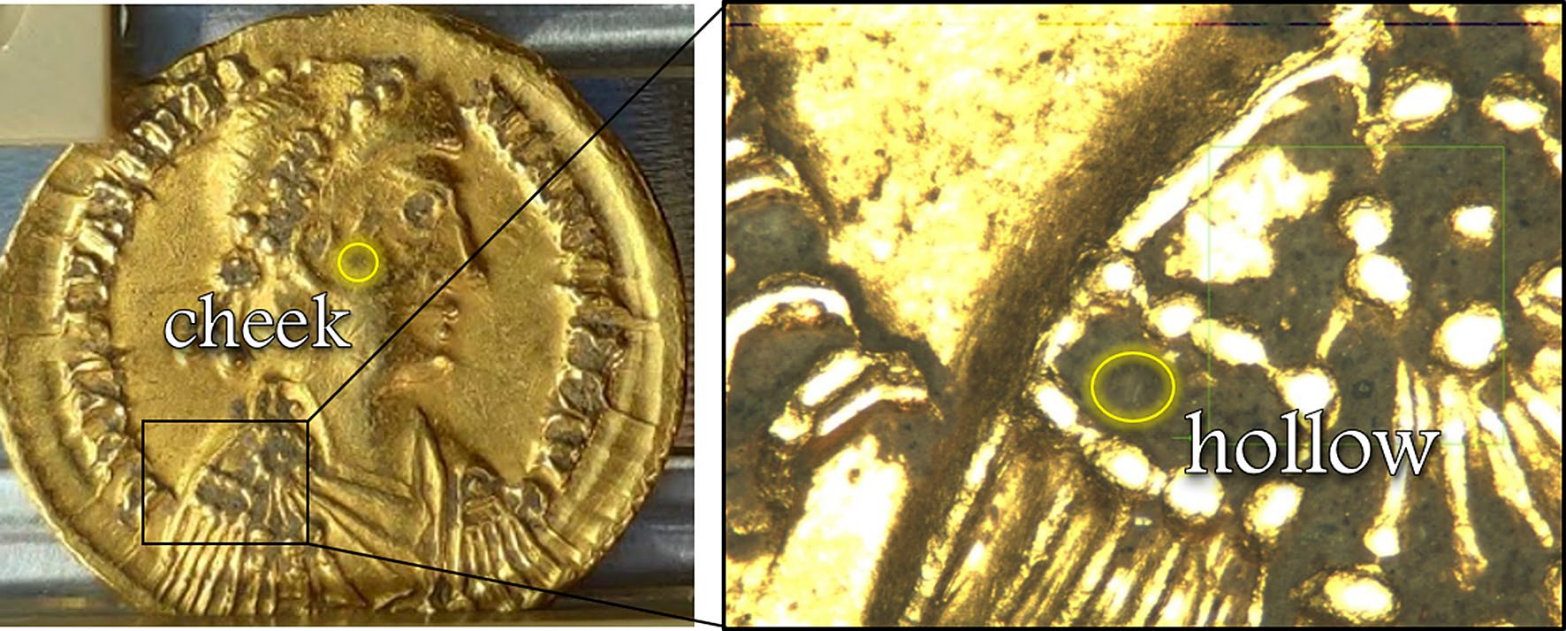
Image of coin D and magnified area showing the two positions where the XANES measurements were carried out.

To gain further insight on the nature of the dirt, the elemental selectivity and chemical sensitivity of XANES were exploited. In particular, we used this technique on coin D to show the possibility of identifying the mineral nature of dirt accumulated on the coin surface. The measurements were carried out at the Fe K-edge in two different areas of the coin: in the cheek area, having an even and rather flat surface, and in a hollow part where some dirt is accumulated; the two positions are shown in Fig. 4.

**Figure 5:**
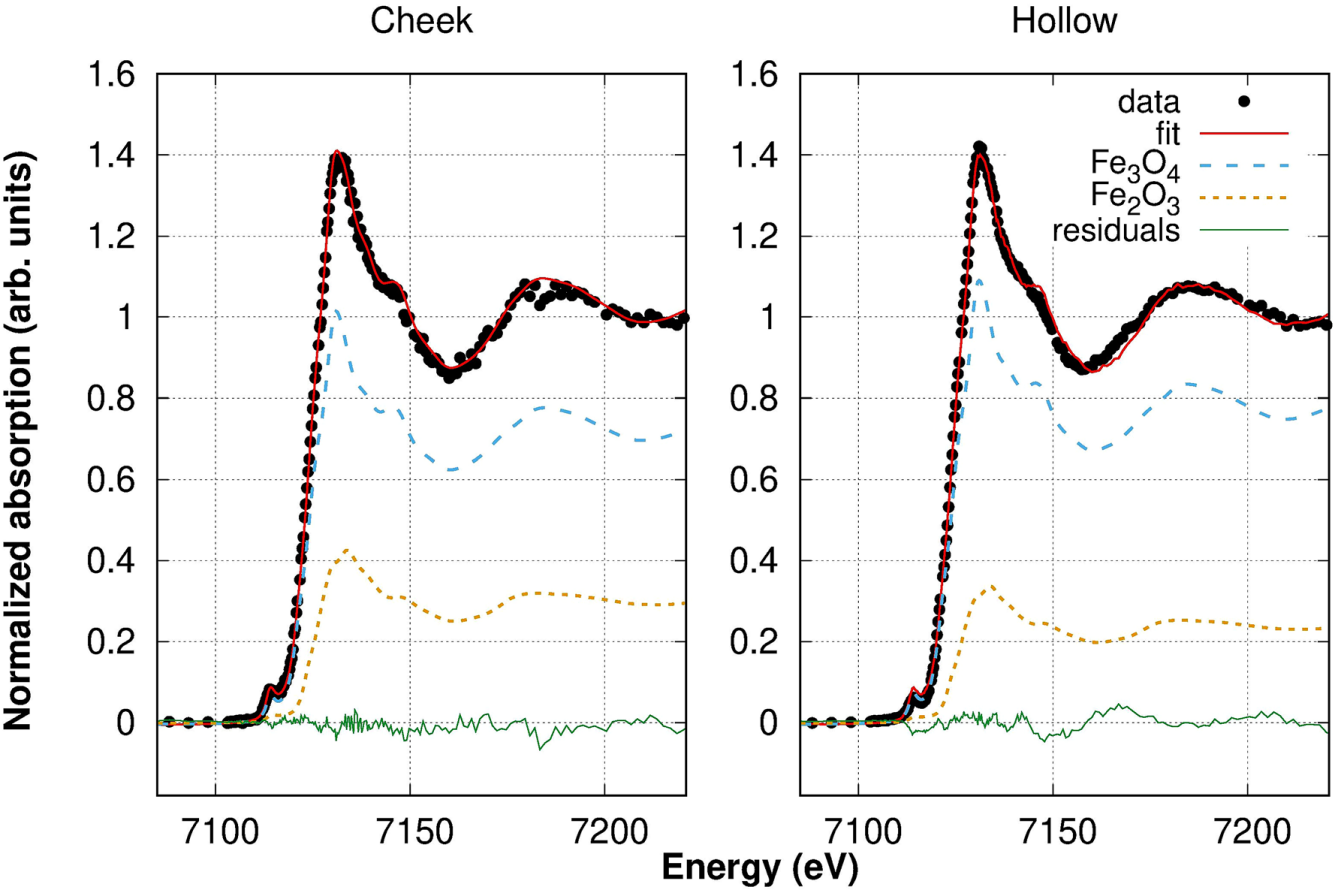
LCF analysis of the Fe K-edge spectra collected on the cheek area (left) and on the hollow area (right) of coin D. In both cases, the contributions needed for the best fit are magnetite (Fe$_3$O$_4$) and hematite (Fe$_2$O$_3$).

The dirt composition was evaluated through the Linear Combination Fitting (LCF) tool available in the Athena software^21^. LCF consists in combining spectra of Fe references to reproduce the experimental spectrum of the sample. The spectra of both regions were satisfactorily reproduced using two contributions: magnetite and hematite, as reported in Table 4. The addition of other phases to our combination (e.g. goethite and wüstite), did not improve significantly the agreement between fit and data shown in Fig. 5. The reference spectra considered are shown in the Supplementary Materials (Fig. S3). We must stress here that in our first attempts of fitting some goethite was found ($<5\%$). However, this amount is lower than the uncertainty of the analysis and, therefore, this minor contribution was excluded.

The small differences between the two regions can be explained considering that the dirt on the flat, more regular surface of the cheek area is expected to be more recent than that found in the hollow region. In facts, the latter, due to its morphology, facilitates the dirt accumulation. The hollow region hosts debris of different times, with the oldest being deeper and the newest being closer to the surface. The outermost layer is expected to be very similar to the clean area, hosting recent debris. Any additional component found in the hollow spectrum and not in the clean one, could be due to a deeper layer of dirt, i.e. to the debris accumulated less recently.

**Table 4:** Relative amount of Fe oxides in the two regions, calculated through LCF analysis of the XANES data. Uncertainties are ±5%.

Fe oxide	Cheek (%)	Hollow (%)
Magnetite (Fe$_3$O$_4$)	71	77
Hematite (Fe$_2$O$_3$)	29	23

The composition of the soil varies depending on the geographical area: for instance, hematite, magnetite, and goethite prevail in tropical and subtropical soils^22^. Typically, the presence of goethite is favoured by the high content of organic matter and by the acid pH of the soils^23^, which usually exhibit a higher affinity for this mineral than for hematite^24^. Higher temperature or lower water activity are probably the most important factors favoring the formation of hematite over goethite^25^. Such conditions are commonly met in tropical environments but might be found also in temperate regions presenting a well-drained calcareous gravel^26^.

According to Ref^5^, the composition of the dirt found on the coin - i.e. magnetite (Fe$_3$O$_4$, with Fe$^{2+}$ and Fe$^{3+}$) and hematite (Fe$_2$O$_3$ with Fe$^{3+}$) - can be related to warm climates (favouring Fe higher oxidation states) and to sandy and silty soils.

This suggests that the burial soil belongs to the Mediterranean region, possibly in the coastal area. Although the exact location where the coins where found is unknown, the pieces come from the Balkan area, in agreement with our results. Comparing the compositions of the two different area, a lower concentration of hematite is observed in the hollow region. As the presence of hematite increases with the mean annual temperature of the environment^26^, this finding can indicate that the outermost layer of dirt comes from a more temperate area than the outer layer, i.e. closer to the coastal region.

## Conclusions

We characterized four gold coins dated back to the V century A.D. using a non-invasive approach combining complementary investigation techniques coupled with statistical analysis tools.

Applying the t-SNE algorithm to spatially resolved XRF data sets, we could identify the elements used in the minting of each piece, disentangling the fluorescence emission of the dirt accumulated on the surface from the signal of the elements making up the original coin.

A semi-quantitative fitting of the XRF spectra relative to the metallic alloy evaluated the Au fineness and the presence of minority alloy elements. Our data shows a variation in the alloy purity of about 2% among the pieces analysed, which is in agreement with the available literature. The lower fineness of some of the coins can be explained with the use of Au alloys probably coming from tax collection as heterogeneous source material of minting. The fine evaluation of the trace elements highlighted a different Pt content in one of the coin (sample A) with respect to the others. This suggests that a different gold source was used to struck this coin with respect to the other samples.

The same quantitative analysis was used to shed light on the nature of the dirt accumulated in an indented area of one coin. In this area, XANES measurements were carried out at the K-edge of Fe, one of the major elements present in the compound. The XANES spectrum collected in a flat, even region showed a weaker signal compared to that collected in a hollow area. What is more, we were able to reproduce the spectral features using LCF with two Fe oxides: hematite and magnetite. From the quantification of these contributions we could identify the environmental conditions compatible with the terrigenous debris accumulated at the very surface (flat, even surface) and in deeper regions (indented part). Our results suggest that the both deposits are consistent with the Mediterranean area, and that the most recent deposits are from warmer, more temperate regions (likely closer to the coastal region) compared to the soil accumulated deeper.

Our approach could be easily and efficiently applied when dealing with encrustation, corrosion, and conservation in general, which are common topics in the field of archaeometry, including numismatics^20^.

## Methods

### Samples

The four specimens were selected from the collection available at the Department of Humanities (DiSU) of the University of Trieste. At the best of our knowledge, the coins were retrieved in the Balkan area. Throughout the text, they are referred to as Coins A, B, C, and D, as given in Table 5. The coins were dated on the basis of the emperor pictured on their obverse: the estimated year is the one marking the middle of the reign period of that emperor. The dating was carried out upon comparison with information by one of the main references of the field^27^. The dating is reported in the first row of Table 2. Samples A and B are dated the same but A was minted in Ravenna (mintmark COM), B in Constantinople (mintmark CON)^28^. Sample C was minted under emperor Theodosius II and is a *tremissis*, i.e. worth 1/3 of a *solidus*. Finally, sample D was chosen to represent a later production, roughly 30 years after the first two coins.

**Table 5:** Details of the four coins analysed in this work. The estimated year is the one marking the middle of the reign period of the emperor pictured on the obverse of the artefact.

Sample	Period (AD)	Coin	Mint (mark)	Emperor
A	409	Solidus	Ravenna (COM OB)	Arcadius
B	409	Solidus	Constantinople (CON OB)	Arcadius
C	417	Tremissis	Constantinople (CON OB)	Theodosius II
D	440	Solidus	Ravenna (COM OB)	Valentinianus III

**Figure 6:**
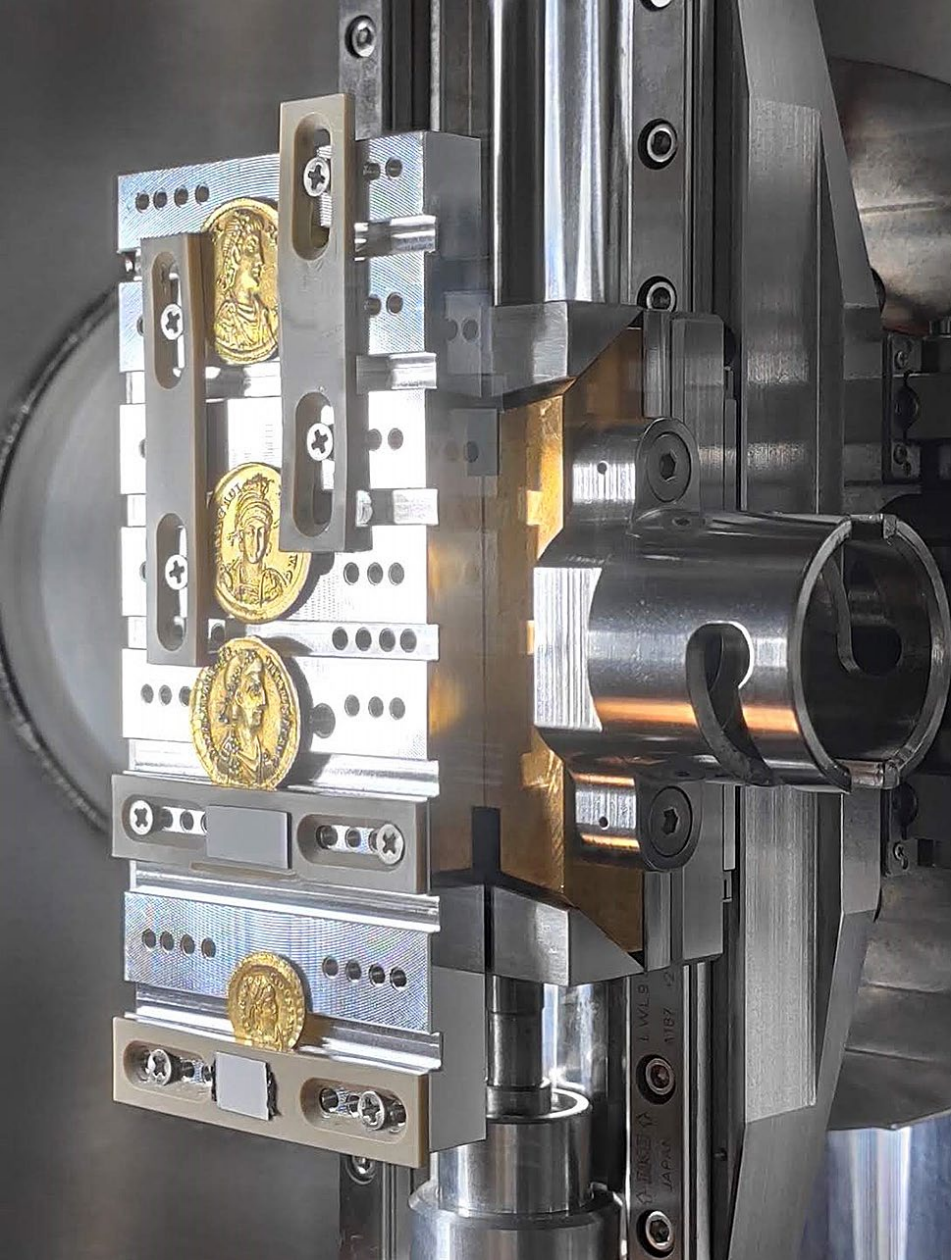
The four gold coins analysed in this work, inside the UHV chamber at the XRF beamline at Elettra Synchrotron.

Before the measurements, the samples were gently brushed to remove dust and coarse dirt deposited on the surface, no chemicals were used in this process. The coins were then mounted on a sample holder using teflon stripes, as shown in Fig. 6. This rough cleaning was aimed at avoiding that coarse dirt fall in the UHV chamber during the measurements. The debris accumulated in the indentations is resistant to gentle brushing using a soft brush, as shown by the magnified images of the samples showing the dirt in the hollow parts even after the brushing. This allowed us to carry out analysis on the accumulated debris and to propose hypotheses on the provenance of the soil.

### Synchrotron radiation characterization

XRF and XANES measurements were carried out in a UHV chamber^29^ at the XRF beamline^3^ with a beam size of $100 \times 100 \upmu \mathrm{m}^2$. The beam intensity before the sample, $I_0$, was monitored using an AH501B diamond detector developed by the detector group of Elettra Sincrotrone Trieste. The system is based on a 4-channel solid state sensor composed of a 12 $\mu$m-thick, free-standing, polycrystalline diamond plate (Dectris, Rigi). Its total active area is 9mm x 3mm subdivided into four electrodes of 4.5 mm x 1.5 mm area each. The individual currents from the four sensors are registered by a 4-channel picoammeter. Every step of the analysis described in this work was carried out after normalizing the raw data for the I$_0$. XRF maps were collected using two exciting energies: below and above the Pt L$_3$ edge, at 10 and 11.6 keV, respectively. The 11.6 keV beam provides an optimal sensitivity to the Pt L lines but the tails of these peak attenuate the signal of the Zn K alpha line, hindering a reliable topography assessment of this element. For this reason, the 10 keV maps were collected. In the analysis, they were used whenever the elemental analysis of the debris was involved. Once the Zn absence in the alloy was proven, the 11.6 keV spectra could be used in the quantification analysis of the gold alloy, taking advantage of the high sensitivity to Pt. Furthermore, XRF measurements carried out on imprinted surfaces of ancient coins at 11.6 keV were proven to be highly reliable and comparable to the same measurements carried out on a polished flat side of the coins^30^. Both energies result into a penetration depth of about $10\ \mu$m but we stress here that the probed depth, limited by the low penetration depth of low-energy X-rays emitted from the sample, is around 1 $\mu m$. The samples were raster scanned using a motorized sample holder and the fluorescence yield was recorded by a single element Silicon Drift Detector (XFlash 5030, Bruker Nano GmbH, Germany) in 45$^{\circ }$/45$^{\circ }$ geometry. Reference metallic foils (purity 99.97%) from Goodfellow were measured in the same conditions and served as calibration standards for the analysis: these included Au, Cu, Fe, Pt, Zn.

XANES spectra were collected at the K-edge of Fe in fluorescence geometry using the same experimental set-up described for XRF measurements. The monochromator was calibrated on the Zn K edge measured on a metallic Zn foil. The data was normalised using standard methods for XANES and using the Athena software^21^.

### t-SNE

The t-SNE algorithm is a multivariate analysis which evaluates independently each pixel in a given XRF map and returns a 2D plot in which each point represents a pixel of the map. This allows to obtain a simpler representation of high-dimensional data in 2D. The raw data was normalised to the I$_0$, then, the t-SNE algorithm was applied to the 10 keV maps: Fig. 2a shows the 2D plot obtained for coin B. The distance between the different points in the 2D plot is an indicator of the spectral similarity of the different pixels, with a shorter distance denoting a higher similarity. However, as density in high dimension is not preserved in the t-SNE plot, data segmentation should be carried out not only on the distribution of the pixels in the 2D plot, but also, simultaneously, on the basis of the average raw spectra of each group and the position of the relative pixels in the original XRF map.

### PyMCA

The spectra were analysed using PyMCA, choosing the SNIP algorithm for background subtraction and the Pseudo-Voigt function for fitting the experimental curve. The fit procedure relied on the fundamental parameters approach, whose values were first refined on the reference compounds and then applied to the specimens. In particular, fundamental parameters were optimised on reference spectra collected on metallic foils of Fe, Co, Cu, Zn, Au, Ag. The relative amount of elements present in the alloys was calculated excluding those elements whose signal was stronger in the “indentation” group.

## Supplementary Information

Supplementary Information.

## Data Availability

All data generated or analysed during this study are included in the supplementary files related to this published article.
