# Supplementary material for: Combining synchrotron radiation techniques for the analysis of gold coins from the Roman Empire
Source: Sci Rep. 2022 Sep 23;12:15919. doi: 10.1038/s41598-022-19682-8 (PMC9508250; doi:10.1038/s41598-022-19682-8)
Supplement: Supplementary file 1 — Supplementary Information. [file 41598_2022_19682_MOESM1_ESM.pdf]

Combining synchrotron radiation techniques for the analysis of gold coins from the Roman Empire

I. Carlomagno[1,*], P. Zeller[1,†], M. Amati[1], G. Aquilanti[1], E. Prenesti[2], G. Marussi[3], M. Crosera[3], and G. Adami[3]

[1]Elettra Sincrotrone Trieste, Trieste, 34149, Italy
[2]Dipartimento di Chimica, Università di Torino, Italy
[3]Dipartimento di Scienze Chimiche e Farmaceutiche, Università di Trieste, Italy
[*]ilaria.carlomagno@elettra.eu
[†]present address: Helmholtz-Zentrum Berlin für Materialien and Energie GmbH, BESSY II, Germany and Fritz-Haber-Institut der Max-Planck-Gesellschaft, Dept. Inorganic Chemistry, Berlin, Germany

ABSTRACT

Four gold coins minted in the V century have been studied with non-destructive synchrotron radiation techniques, namely X-Ray Fluorescence (XRF) and X-ray Absorption Near Edge Spectroscopy (XANES). XRF data analyzed coupling standard and statistical methods were used to distinguish the composition of the alloy constituting the coins from that of successive deposits processes. Our analysis presents a quantification of the trace elements present in the metallic alloy providing interesting details for historical insight. Furthermore, on the basis of the XRF maps, some regions of interest were selected for XANES at the K-edge of Fe. Our analysis of the Fe spectra points out two main phases which can be related to Fe oxides naturally present in soil. From the relative abundance of these oxides, information on the site where the coins were found can be obtained, providing additional information on their fate across the centuries.

1 Supplementary Material

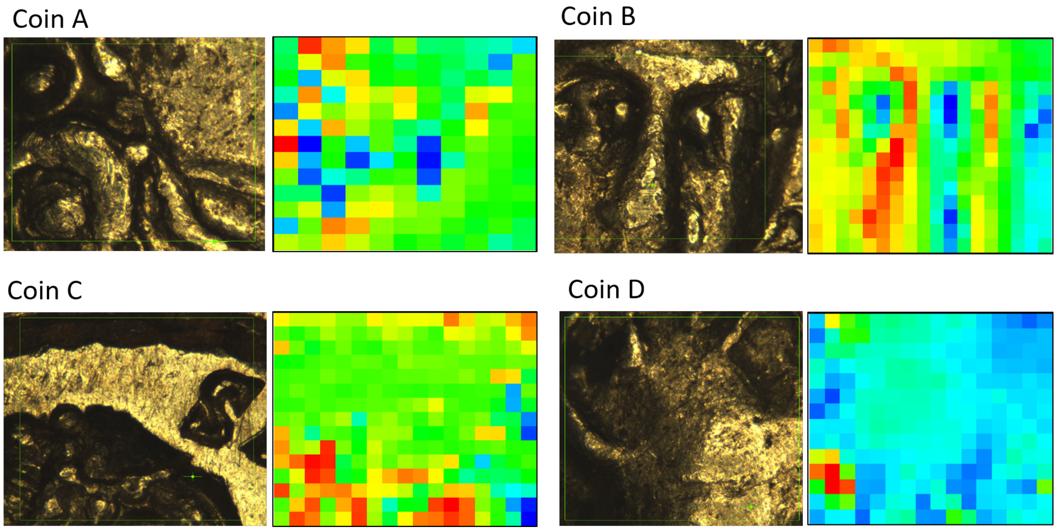

Figure S1. XRF maps collected on the four coins under investigation.

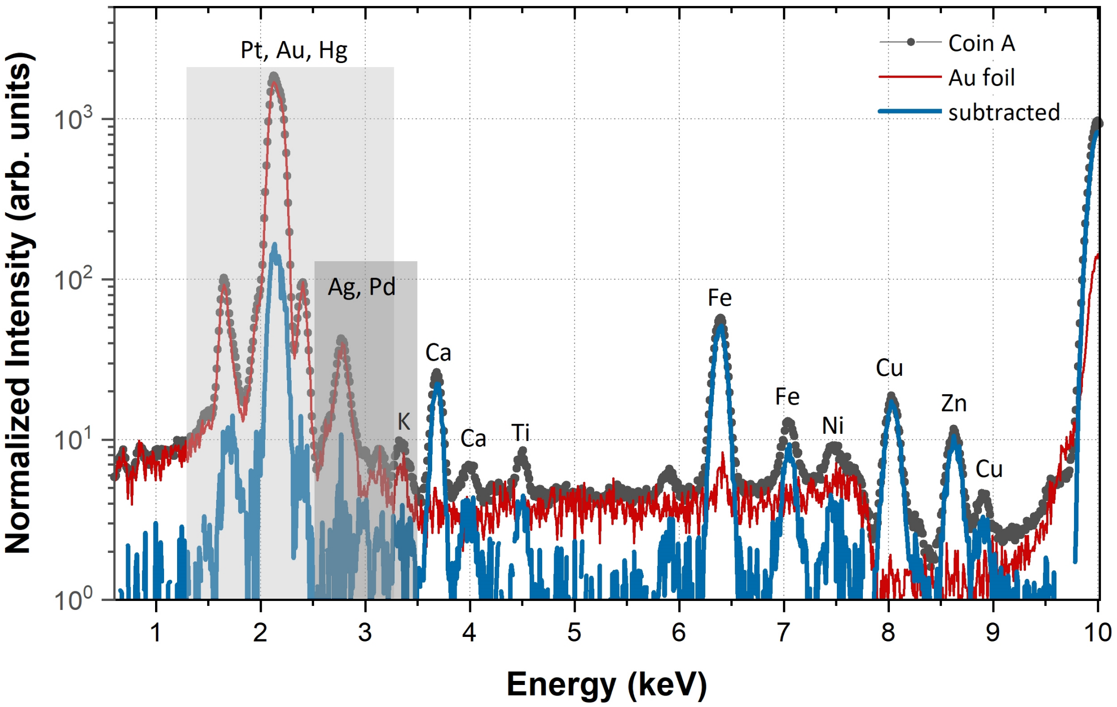

Figure S2. XRF spectra collected at 10 keV on Coin A (black points) and on an Au foil (thin red line). The difference of the spectra is reported as thick blue line. Labels identify the fluorescence emission of the elements: the elements reported twice refer to Kα and Kβ lines.

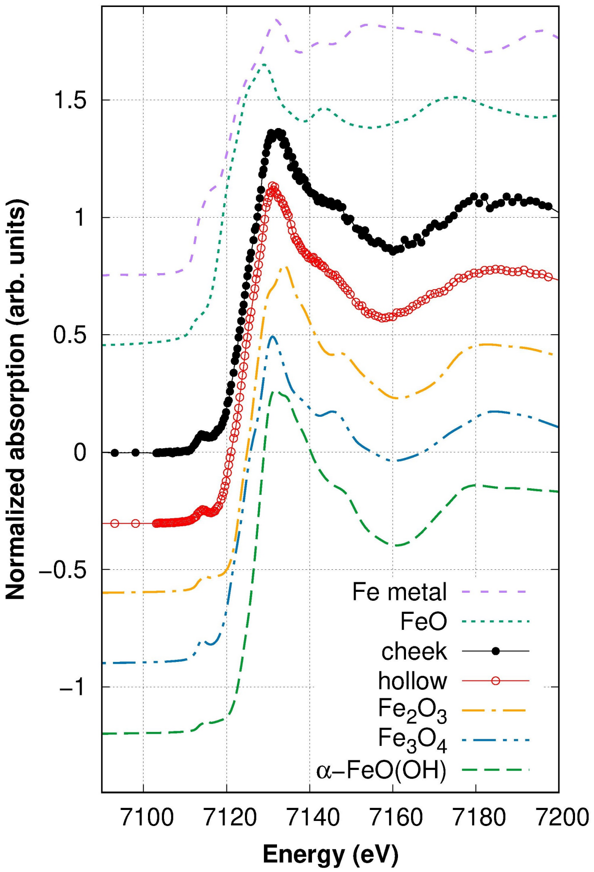

Figure S3. The points show experimental XANES spectra collected in the cheek and hollow areas of sample D (solid and hollow points, respectively). Dashed lines present the reference spectra used for the linear combination fitting (LCF) as discussed in Sec. 1.4 , and additional reference spectra of a Fe foil and of the wüstite phase (FeO). The spectra are shifted for clarity.
